# Glutamatergic dysfunction of astrocytes in paraventricular nucleus of thalamus contributes to adult anxiety susceptibility in adolescent ethanol exposed mice

**DOI:** 10.1038/s41386-025-02264-3

**Published:** 2025-10-13

**Authors:** Aubrey Bennett, Hyunjung Kim, David Thomas, Peter Biggs, Roxan Ara, Asamoah Bosomtwi, Seungwoo Kang

**Affiliations:** 1 Department of Pharmacology and Toxicology, Medical College of Georgia, Augusta University, Augusta, GA USA; 2 Georgia Cancer Center, Medical College of Georgia, Augusta University, Augusta, GA USA; 3 Department of Biochemistry and Molecular Biology, Medical College of Georgia, Augusta University, Augusta, GA USA

**Keywords:** Astrocyte, Cellular neuroscience, Emotion

## Abstract

Repeated ethanol exposure during adolescence increases adult anxiety risk, but the underlying mechanisms remain unclear. The paraventricular nucleus of the thalamus (PVT) has been considered a hub for controlling anxiety and is affected by experiences from early life. Thus, this study investigated how adolescent intermittent repeated ethanol exposure (AIE) affects the PVT activities and anxiety-related behaviors in adulthood. We found that AIE triggers anxiety-like behaviors and parallelly exhibited elevated firing rates and increased calcium signaling in the PVT neurons compared to control counterpart mice. Chemogenetic inhibition of PVT neurons reduced anxiety-like behaviors in AIE-treated animals, confirming PVT’s role in adolescent alcohol-induced adult anxiety. The increased PVT neuronal activities were mediated, at least partly, by the reduced GLT1, an astrocyte dominant glutamate transporter (also known as EAAT2, slc1a2). Magnetic resonance spectroscopy showed the higher glutamate/GABA ratios in the thalamus of GLT1 knockdown mice, which also exhibited heightened anxiety-like behaviors. Importantly, the selective GLT1 deletion in the PVT astrocytes of alcohol-naïve mice elicited anxiety-like behaviors, whereas GLT1 enhancement in PVT astrocytes of AIE-treated mice ameliorated AIE-induced anxiety-like behaviors. These findings highlight the significant role of PVT astrocytic GLT1 in the anxiogenic phenotype in adulthood induced by adolescent intermittent ethanol exposure, suggesting that GLT1 in the PVT could serve as a therapeutic target for alcohol use disorder and comorbid emotional disorders.

## Introduction

Adolescence represents a critical neurodevelopmental period for brain maturation processes, including the establishment of mature neurotransmitter systems [1–4]. As a result, those who are exposed to alcohol at this early period are more likely to experience many psychological and physical issues even into adulthood [5–13]. One remarkable consequence of exposure to alcohol on the adolescent brain is the fact that it increases the likelihood developing generalized anxiety disorder later in life [14–18], which in adults is also associated with alcohol use disorder (AUD) [19]. AUD and anxiety are indeed not only common comorbidities but also contribute to each other’s development [13, 19]. However, the neurobiological mechanisms underlying adolescent chronic intermittent alcohol exposure-induced adult behavioral susceptibility in anxiety-like behavior remain unclear.

The thalamic circuits mediating arousal have been recognized as a link between the adolescent experience and the molecular and behavioral traits associated with psychiatric disorders in adults [20–23]. Especially, the paraventricular nucleus of thalamus (PVT) has been considered a hub of anxiety network [24, 25] because anxiety is characterized as a state of alertness and hypervigilance to external environmental cues [26]. Indeed, adverse experiences in early life induce the pathophysiological adaptation in the PVT, and consequent anxiogenic profiles in rodent models [23, 27]. However, it remains unknown if the repeated ethanol exposure during adolescence will affect the coordinated brain activities of the PVT in adulthood, and consequent behavioral adaptation.

Chronic intermittent ethanol exposure is frequently accompanied by pathological hyperglutamatergic states in the brain, characterized by sustained elevations in extracellular glutamate concentrations and neuronal vulnerability to excitotoxic damage and aberrant synaptic transmission [28]. Under normal physiological conditions, astrocyte dominant glutamate transporters, including GLT1 and GLAST (also known as EAAT2 and EAAT1, respectively), maintain precise glutamate homeostasis by rapidly scavenging excess glutamate from tripartite synapses, thereby preventing excitotoxicity and providing optimal synaptic conditions for subsequent synaptic events [28]. However, chronic alcohol exposure frequently affects this tripartite synaptic regulation, resulting in decreased transporter expression, impaired glutamate uptake capacity, and consequent accumulation of extracellular glutamate [29–34]. Given the role of astrocytic glutamate transporters in brain development throughout adolescence [35] and that this adolescent period coincides with increased vulnerability to alcohol use [36], it is intriguing to evaluate whether adolescent intermittent repeated exposure to ethanol may produce particularly pronounced and lasting disruptions to glutamate homeostasis. Furthermore, since adolescent brains are much more adaptable than adult brains, heightened neuroplasticity during the adolescent period may render glutamate transporter systems more susceptible to ethanol-induced modifications, potentially establishing maladaptive patterns that persist into adulthood. This possibility prompted us to examine the specific effects of ethanol exposure on astrocytic glutamate transporter function and expression in adult mice exposed to alcohol repeatedly during the adolescent period. Our particular attention is on how these changes might contribute to the increased susceptibility to anxiety-like behaviors, which is one of the main AUD comorbidities [37–39].

In the current study, using chemogenetics, electrophysiology, calcium imaging, magnetic resonance spectroscopy (MRS), and behavioral evaluation approaches with transgenic mouse models, we revealed how the AIE leads an adaptation in glutamatergic signatures via the astrocyte-neuron interaction in the PVT and a subsequent behavioral adaptation.

## Materials and methods

### Animals

All experimental procedures received approval from the Augusta University Institutional Animal Care and Use Committee and were conducted in accordance with NIH guidelines. C57BL/6 J mouse line (Catalog no. 000664) and the GFAP^cre/+^ line [Catalog no. 024098; B6.Cg-Tg(Gfap-cre)77.6Mvs/2 J] were acquired from Jackson Laboratory (Bar Harbor, ME). GLT1-flox mice (Catalog no. 026619; B6.Cg-Slc1a2^tm1.1Ncd^/J) [40] were received from Drs. Doo-Sup Choi at Mayo Clinic and Niels Christian Danbolt at University of Oslo. Ai9-GLT1 mice for cre-dependent overexpression of GLT1 were received from Drs. Doo-Sup Choi at Mayo Clinic and Ho Lee at Korea Cancer Center. Mice were accommodated in standard Plexiglas cages. The colony room was regulated to a consistent temperature of 24 ± 1 °C and humidity of 60 ± 2%, with a 12-h light/dark cycle (lights on at 07:00 a.m.). Mice were provided *ad libitum* access to food and water. Since accumulative rodent studies showed that early adolescent intermittent ethanol (AIE) exposure induces anxiety-like behaviors in adulthood regardless of sex [41, 42], in this study, we used both male and female mice.

### Stereotaxic surgery for virus injection

Mice were anesthetized with isoflurane (1.5% in oxygen) and positioned on the rotational digital stereotaxic apparatus (RWD Life Science). The skull was aligned with a dual-tilt position equalizer and holes were drilled in the skull at the designated stereotaxic coordinates. Viruses were infused to the posterior parts of the PVT (AP −1.6 mm, ML + 0.0 mm, DV −3.0 mm from bregma) at a rate of 100 nl/min for 3 minutes via a 34-gauge needle (Catalog no. NF34BV; World Precision Instruments) with a micro-syringe pump (World Precision Instruments). The injection needle was kept in place for an extra 10 min following the injection. We injected the AAVs at the following titers: AAV_5_-CaMKIIa-GCaMP6s, 4.7 × 10^12^ GC/ml (UNC Vector Core); AAV_5_-CaMKIIa-hM4Di-mCherry, 2.4 × 10^13^ GC/ml (Addgene); AAV_5_-GFAP-mCherry, 3.1 × 10^12^ GC/ml (Vector Biolab); AAV_5_-GFAP-mCherry-Cre, 4.3 × 10^12^ GC/ml (UNC Vector Core). We administered buprenorphine sustained release (1.3 mg/kg, s.c.; CoVetrus) to mitigate postoperative pain.

### Brain slice preparation and ex vivo electrophysiology

Brain slices containing the PVT region were prepared for electrophysiological recordings, as described [43]. Briefly, mice were deeply anesthetized by isoflurane inhalation, after which the brain was rapidly extracted and immersed in ice-cold sucrose-based artificial cerebrospinal fluid (aCSF) containing the following (in mM): 87 NaCl, 75 sucrose, 2.5 KCl, 11.25 NaH_2_PO_4_, 0.5 CaCl_2_, 7 MgCl_2_, 25 NaHCO_3_, 0.3 l-ascorbate, and 25 glucose, and oxygenated with 95% O_2_/5% CO_2_. Coronal brain slices (300–350 μm) were cut with a vibrating compresstome (VF-310-0Z, Precisionary Instruments), subsequently placed in a slice holding chamber and incubated for 30 min at 34 °C and kept for at least 1 h at room temperature (24–25 °C) in carbonated (95% O_2_/5% CO_2_) standard artificial cerebrospinal fluid (aCSF) containing the following (in mM): 126 NaCl, 1.25 NaH_2_PO_4_, 1 MgCl_2_, 2 CaCl_2_, 2.5 KCl, 25 NaHCO_3_, and 11 glucose. Electrical signals were captured using an Axon 700B amplifier, a Digidata 1550B A/D converter, and Clampfit 11.0 software (Molecular Devices). Throughout the experiments, the bath was consistently perfused with warm (32 °C) carbonated aCSF at a rate of 2.0–2.5 ml/min. Patch pipettes had a resistance of 4–6 M*Ω* when filled with a solution containing (in mM): 140 Cs-methanesulfonate, 5 KCl, 2 MgCl_2_, 10 HEPES, 2 MgATP and 0.2 Na_2_GTP for voltage clamping. The pH was adjusted to 7.2 with Tris-base and the osmolality to 310 mOsmol/L with sucrose. Healthy cells were identified using a high magnification microscope (at 400×, Nikon FN1 Microscope, Melville, NY) based on their morphology (round, ovoid, and non-swelled plasma membrane). The spontaneous firing was recorded using the loose-patch cell-attached method.

### In vivo electrophysiology

The in vivo electrophyiological recordings were performed as described [43, 44]. Briefly, mice were anesthetized by urethane (1.5 g/kg, *i.p*.; Sigma–Aldrich) [43] and positioned on a stereotaxic frame (RWD Life Science). We continuously monitored respiration rate and pedal withdrawal response during recordings, while the body temperature was maintained using a small-animal feedback-controlled warming pad (Kent Scientific Corporation). Small burr holes on skulls were created to insert high-impedance 32-channel microelectrodes (H10b, Cambridge NeuroTech). The reference wire (Ag/AgCl, 0.03 inches in diameter, A-M systems) was positioned in the contralateral parietal cortex. Electrophysiological signals were digitized at 20 kHz and band-pass–filtered from 300 to 3000 Hz (Intan Technologies). We verified single-cell spiking during the recording by checking spiking shapes using spike scope and comparing event timings using spike filter display in the Intan RHX Data Acquisition Software (Intan Technologies). The data were analyzed with Clampfit (version 11.2, Molecular Devices) and a custom-written code in MATLAB (R2019a, The MathWorks).

### In vivo Ca^2+^ signal with fiber-photometry

We monitored cellular Ca^2+^ transients in real-time in vivo by fiber-photometry as described [15, 44]. Three weeks post-injection of the AAV encoding GCaMP6s in the PVT, we implanted a fiber optic cannulae into the PVT (AP −1.40 mm, ML + 0.0 mm, DV −2.8 mm from bregma). The fluorescence signals were captured at 30 frames per second using a multi-wavelength fiber-photometry system (Plexon Multi-Fiber Photometry System, Plexon, Dallas, Texas), which had a dichroic mirror and a lens link to a photomultiplier tube (PMT) to reflect beams from 465 to 410 nm LED wavelengths. We used 410 nm as an isosbestic control to correct for calcium-independent fluorescence, movement artifacts, and photobleaching, 465 nm for the excitation of GCaMP6s. The collected data were analyzed using customized Mat-Lab codes and MatLab-based photometry modular analysis program, pMAT [45]. The ΔF/F is generated by subtracting the fitted isosbestic control (410 nm) from the signal (465 nm) to eliminate movement or other common artifacts. Then, those calcium transients were normalized as a Z-score. The Z-scores of spatiotemporal calcium transients were then sorted and averaged by animal location information (center vs. periphery) within a single animal and reported as the average Z-score across animals [46, 47].

### Chemogenetics and drug treatments

Dreadd ligand, JHU37160, was purchased from Hello Bio (J60; JHU37160 dihydrochloride, Cat No. HB6261, Princeton, NJ), noted for its superior blood-brain barrier penetrance and enhanced affinity, potency, and selectivity of DREADDs (Designer Receptors Exclusively Activated by Designer Drugs) [48]. For the activation of DREADDs, we administered J60 [0.3 mg/kg, dissolved in the vehicle, 1× phosphate-buffered saline (PBS)] intraperitoneally 10 min prior to the experiments in mice. The concentration has been demonstrated to have no off-target effects in rodent behavioral tests [48–52].

### Magnetic resonance spectroscopy (MRS)

All experiments were conducted using a Bruker Biospec 7.0 Tesla 30 cm horizontal bore scanner (Bruker Biospin MRI GmbH, Germany), featuring a BGA12SHP gradient system that generates pulse gradients of 660 mT/m along each of the three axes, equipped with AVANCE III HD electronics and interfaced to a Bruker Paravision 6.0.1 console. A 4-channel surface phase array coil (Rapid MRI International, Columbus OH) served as the receiver, while a Bruker 86 mm linear-volume coil as the transmitter. Throughout the recordings, the animal was maintained under 1–2% isoflurane anesthesia and respiratory rate and body temperature were consistently monitored. The total duration of the whole imaging experiment was approximately 1.5 h. For 1H MRS, adjustments of all first- and second-order shims within the voxel of interest were accomplished with the MAPSHIM procedure. The in vivo shimming procedure yielded a full width half maximum (FWHW) ranging from 7.8 to 10.9 Hz for the unsuppressed water peak in the spectroscopic voxel of the mouse brain. The water signal was attenuated by variable power radiofrequency (RF) pulses with optimized relaxation delays (VAPOR). Outer volume suppression (OVS) integrated with a point-resolved spectroscopy (PRESS) sequence was employed for signal acquisition, utilizing 2 × 2 × 2 mm^3^ voxel, with a TR/TE = 2500/20 ms, a spectral bandwidth of 4 kHz, 2048 data points, and 256 averages. Spectral data were acquired from the voxel encompassing the dorsal thalamic brain region including the PVT according to the mouse brain atlas. Metabolite quantification was conducted utilizing the LC-Model (version 6.3-1 L) alongside the spectral basis set provided by the vendor. The absolute concentrations of the following metabolites were assessed: aspartate, creatine, γ-aminobutyric acid (GABA), glutamine, glutamate, guanidinoacetate, glycerophosphorylcholine, lactate, myo-inositol, N-acetylaspartate, N-acetylaspartylglutamate, Phosphocreatine, phosphorylcholine, and taurine. Only those results with Cramér-Rao lower bounds (CRLB, % SD)  ≤  50% were included in the statistical analysis.

### Repeated intermittent ethanol exposure

**Fig. 1:**
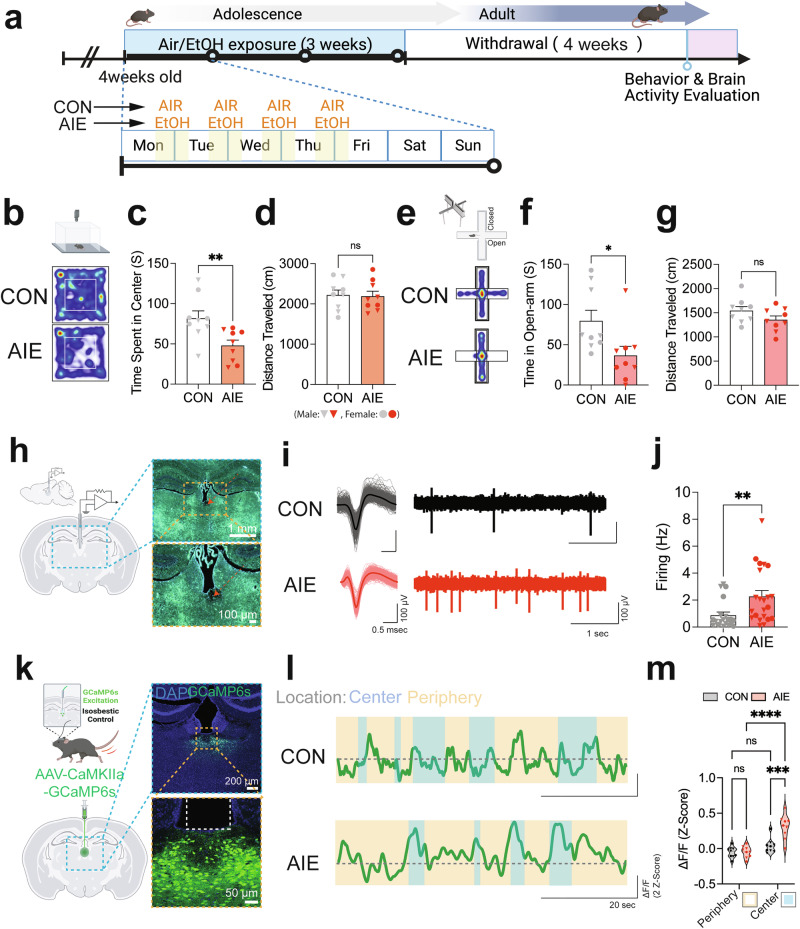
Adolescent repeated ethanol exposure (AIE) induces anxiety-like behaviors in adult mice and increased neuronal activities in the PVT. Diagram to explain the experimental procedure (**a**). Representative traces (**b**, **e**) and pooled data (**c**, **d**, **f**, **g**) showing that adult mice at 4 weeks withdrawal from repeated ethanol exposure during adolescent period (AIE) show heightened anxiety-like behaviors compared to air-exposed counterpart mice (CON) in open field test (**b**–**d**) and elevated plus maze test (**e**–**g**). Diagram and representative traces (**h**, **i**) and pooled data (**j**) showing that increased spontaneous firing of PVT neurons after AIE. Representative expression of GCaMP6s in the PVT (**k**), behavior-synced calcium traces (**l**), and pooled data (**m**) showing that the calcium transients in the PVT neurons are significantly increased in the AIE group when exposed anxiogenic behavioral task. Female (circles) and male (triangles). Data represented as mean ± SEM. *p < 0.05, **p < 0.01, ***p < 0.001, ****p < 0.0001. Fig. 1a, b, e, h, and k were created using BioRender.com.

The repeated intermittent ethanol exposure procedure has been described [53]. Mice were subjected to ethanol exposure in a vapor inhalation chamber [54] for three weeks, from postnatal day 28 to postnatal day 46. Each daily cycle included either 16 h of ethanol vapor (AIE group, BEC 100–150 mg/dL at the end of the session) or equivalent counterpart room air (CON group), followed by 8 h of abstinence in their home cage away from both vaporized ethanol and air. This was repeated daily for four consecutive days, succeeded by three days of abstinence. The cellular activity and animal behaviors were assessed 4 weeks post-AIE procedure (Fig. 1a and Supplementary Fig. 1a).

### Behavioral evaluations

All home cages housing mice were transported to the behavior testing room a minimum of 2 h before the testing start. The anxiety-like behaviors and spatial working memory were evaluated, and all the behaviors were assessed only during the light phase between 12 p.m. and 4 p.m. (Supplementary Fig. 1). The tests were performed in the same order; the mice underwent open field test (OFT) and elevated plus maze test (EPM). In the case of additional evaluations in light-dark test (LDT) and Y-maze, the mice underwent LDT and Y-maze. The behavioral experiments were conducted every other day during the same period of the day, because the period of the day can also have an impact on the behavior. The order of the animals was randomized. All the tests were scored using video tracking software, Ethovison (Ethovision XT, Noldus).

#### Open field test

The OFT was performed in a chamber (40 × 40 × 40 cm) to assess anxiety-like behaviors and locomotion. The session went for 30 min in the testing room under low light intensity (~30 lux) and the duration in the center area (25 × 25 cm) and distance to travel in the initial 5-min served as a reference for assessing anxiety-like behavior.

#### Elevated plus maze test

Elevated plus maze (EPM) was positioned 50 cm above the ground and comprised two open (35 ×6 × 0.5 cm) and two closed (35 × 6 × 22 cm) arms, together with a connecting central zone (6 × 6 cm). Mice were placed in the closed arm of the EPM, facing to the end of the arm. Mice were allowed to navigate the maze for 5 min, during which the duration spent in the open arms and distance traveled velocity were recorded.

#### Light-dark box test

The light-dark box test (LDT) was performed in a chamber designed as a modified open-field arena (60 × 40 × 30 cm), where about two-thirds of the apparatus is brightly lighted (~550 lux) and the remaining one-third is devoid of light (~3 lux). To create the different light intensity in the two areas, the illumination in the testing room was deactivated (0 lux), and LED lights were affixed to the wall separating the two areas, directed towards the brightly illuminated open area. The animal was placed in the non-illuminated section of the apparatus to freely explore both sections for 10 min; time spent in the lit section and distance traveled were recorded as 5-min time bins and the first 5-min bin was used to compare the anxiety levels between groups.

#### Spontaneous alteration behavior in Y-maze

Spontaneous alternation, an assessment of spatial working memory, was conducted in a Y-maze arena (each arm: 30 × 6 × 15 cm). Mice were placed in one arm of a Y-maze, oriented towards the wall, and allowed 10 min to explore all three arms. The alternation ratio was calculated by dividing the number of complete visiting (A to B to C) by the total number of arms entered, subtracting two.

### Western blotting

Tissue containing the PVT was punched out from coronal slices (1 mm thick) and homogenized in ice-cold RIPA lysis buffer (Thermo Fisher) containing a protease inhibitor cocktail (Roche). Equal amounts of protein extracts were denatured and subjected to SDS-PAGE using 4–12% Bis-Tris gels and transferred to PVDF membranes (Thermo Fisher). PVDF membranes were blocked with tris-buffered saline containing 0.05% Tween 20 and 5% (w/v) non-fat dried milk. These membranes were incubated with anti-GLT1 antibody (1:2000, Guinea pig, Millipore Sigma Cat# AB1783, RRID:AB_90949), anti-GLAST antibody (1:2000, Rabbit, Alomone Labs Cat# AGC-021, RRID:AB_2039885), anti-GAPDH antibody (1:2000, Mouse, Sigma Aldrich Cat# MAB374, RRID:AB_2107445), and anti-xCT antibody (1:1000, Rabbit, ABclonal Cat# A2413, RRID:AB_2863004) overnight at 4 °C. After washing with TBST, the membranes were incubated for 1 h with appropriate horseradish peroxidase-conjugated secondary antibodies for 1 h at room temperature. The proteins were visualized by ECL solution (Thermo Fisher) using the G:Box Chemiluminescent Imaging System (Syngene).

### Immunofluorescence

Brains were fixed with 4% paraformaldehyde (Sigma-Aldrich, St. Louis, MO) and transferred to 30% sucrose (Sigma-Aldrich) in PBS at 4 °C for 72 h. Brains were then frozen in dry ice and sectioned at 50 µm using a microtome (Precisionary, Ashland, MA). Brain slices were stored at −20 °C in a cryoprotectant solution containing 30% sucrose (Sigma-Aldrich) and 30% ethylene glycol (Sigma-Aldrich) in PBS. Sections were incubated in 0.3% Triton X-100 (Sigma-Aldrich), 3% bovine serum albumin in PBS for 1 h, followed by incubation with the primary antibody in 3% bovine serum albumin overnight at 4 °C. GLT1 colocalization was examined using primary antibodies against GLT1 (Rabbit, 1:500, Abcam Cat# ab205248, RRID:AB_2924274) with either of S100β (Guinea Pig, 1:500, Synaptic systems Cat# 287004, RRID:AB_2620025) for astrocytes or NeuN (Guinea Pig, 1:500, Synaptic systems Cat# 266004, RRID:AB_2619988) for neurons, followed by corresponding secondary antibodies conjugated with Alexa Fluor 594 (Goat anti-Rabbit IgG, 1:500, Abcam Cat# ab150180, RRID:AB_2650602) and Alexa Fluor 488 (Goat anti-Guinea Pig IgG, 1:500, Abcam Cat# ab150185, RRID:AB_2736871). After three washes in PBS, the sections were mounted onto a microscope slide and cover-slipped with mounting medium (Abcam). Images were captured on an LSM 700 laser scanning confocal microscope (Carl Zeiss) using a 10× or 40× water-immersion lens or a Nikon AX confocal microscope with NSPARC (Nikon) using a 20× lens. The Fluorescent intensity of the images was quantified using Image J (Fiji [64 bit], Image J ver. 1.54p).

### Glutamate assay

For assessing the glutamate levels in the brain tissue, including the PVT, a colorimetric glutamate assay kit was used (Abcam, Cat# ab83389). The optical density (OD) value of each well was measured using a Spectrophotometer (Multiskan FC Photometer, Thermo Fisher) at an absorption wavelength of 450 nm for each assay.

### Data analysis

Data were analyzed by unpaired *t*-tests, one-way and two-way ANOVAs, and post-hoc tests as appropriate and indicated for each experiment. All data are represented as mean ± SEM using Prism 10.1 (GraphPad Software, San Diego, CA). The statistical significance was set at *p* < 0.05. Detailed statistical tests and data with exact *p* values are listed in Supplementary Table 1.

## Result

### The anxiety-like behaviors in adult mice exposed to ethanol repeatedly during adolescent period are accompanied with hyperactivity of PVT neurons

To characterize the animal behaviors and brain adaptation induced by the adolescent chronic intermittent alcohol (ethanol) exposure (AIE) in adulthood, we exposed the mice to ethanol or counterpart air (CON) based on chronic intermittent ethanol exposure procedure when the animals were four weeks old (Fig. 1). The procedure persisted for three weeks and ended before the animals escaped the adolescent period. After four weeks withdrawal from the last ethanol exposure, we subsequently evaluated the anxiety-like behaviors through open filed test (OFT), EPM, and LDT (Fig. 1b–g and Supplementary Figure 1). While the total distance traveled was similar in both groups (Fig. 1d; Unpaired *t*-test, *t* = 0.1698, df = 16, *P* = 0.8673, *N*_mice_ = 9/group), the time spent in the center area in the OFT was significantly shorter in the AIE group compared to that in the CON group (Fig. 1c; Unpaired *t*-test, *t* = 2.966, df = 16, *p* = 0.0091, *N*_mice_ = 9/group). In EPM test, AIE group spent less time than the CON group in the open-arms (Fig. 1f; Unpaired *t*-test, *t* = 2.454, df = 16, *N*_mice_ = 9/group). Likewise, AIE group spent less time than the CON group in the bright light illuminated section during the LDT (Supplementary Fig. 1b–d; Unpaired *t*-test, *t* = 2.173, df = 14, *p* = 0.0474, Nmice = 8/group). Compared to the CON group, the AIE group mice did not exhibit significant changes in short-term spatial working memory in the spontaneous alternation behavior test (SAB) at the 4 weeks withdrawal from AIE procedure (Supplementary Fig. 1e–g; Unpaired *t*-test, *t* = 1.02, df = 14, *p* = 0.3254, *N*_mice_ = 8/group), indicating that repeated alcohol exposure during adolescent period increased the susceptibility to anxiety-like behaviors in adult.

Next, we examined whether the AIE procedure induces adaptation of the neuronal activities in the PVT, an area that has been considered a hub of arousal and anxiety network [24, 25, 55], via electrophysiology and temporal calcium imaging with fiber-photometry. In the in vivo electrophysiology, the basal rate of spontaneous spiking was significantly higher in the PVT neurons of AIE mice than that of CON mice (Fig. 1h–j; Unpaired *t*-test, *t* = 2.738, df = 40, *p* = 0.0092, *N*_cell_ = 20-22 [5 mice/group]). To further clarify whether the AIE-induced anxiety-like behaviors are related to the neuronal activities in the PVT, we measured spatiotemporal cellular activities of the PVT neurons with GCaMP6s, a genetically encoded Ca^2+^ indicator, synched with the behaviors in the OFT (Fig. 1k–m, Supplementary Fig. 2). The results showed that, AIE mice had enhanced calcium transients in the center area compared to that of CON group (Fig. 1k–m, two-way ANOVA F(1,32) = 34.54, *P* < 0.0001 for Group, post-hoc Center:CON vs. Center:AIE: *p* = 0.0001), while the mice did not show any group differences in the calcium transients when they stayed in the peripheral area (Fig. 1m, post-hoc Periphery:CON vs. Periphery:AIE: *p* = 0.9996). These data suggest that the hyperactivity of PVT neurons is accompanied with AIE-induced anxiety-like behaviors.

### Chemogenetic inhibition of the PVT neurons alleviates the anxiety-like behaviors induced by AIE in adulthood

**Fig. 2:**
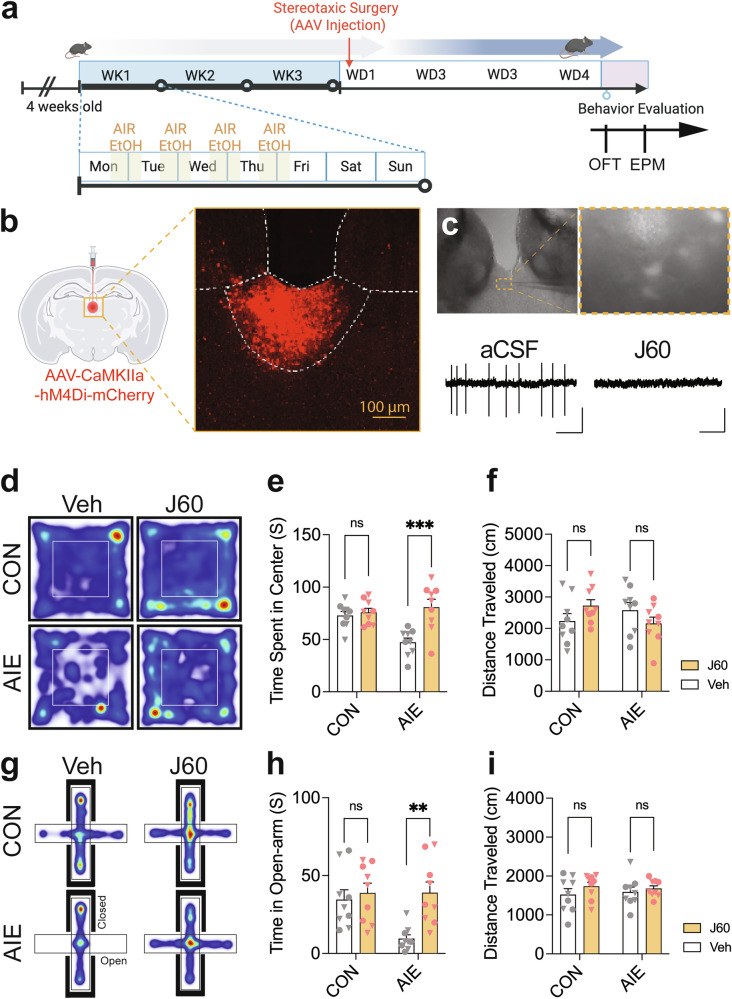
Chemogenetic inhibition of PVT neurons alleviates anxiety-like behaviors induced by AIE. **a**–**c** Diagram of experimental schedules (**a**) and representative figures showing the expression of hM4Di-mCherry in the PVT. (**c**) Representative traces showing the silence of neuronal activity in the hM4Di-positive neurons in the PVT by bath application of DREADDs ligand (J60, 20  $\mu$M). Represenetative traces (**d**, **g**) and pooled data (**e**, **f**, **h**, **i**) showing that the inhibition of PVT neuronal activities by chemogenetic application rescues the anxiety-like behaviors seen in AIE mice in the open filed test (**d**–**f**) and elevated plus maze test (**g**–**i**). Female (circles) and male (triangles). Data represented as mean ± SEM. **p < 0.001, ***p < 0.0001. J60: JHU37160 (0.3 mg/kg, i.p.). Fig. 2a, b, d, and 2g were created using BioRender.com.

To determine whether the PVT neuronal adaptation contributes to the anxiogenic phenotype in adulthood of the AIE mice, we sought to check whether chemogenetic inhibition of the PVT neurons will ameliorate the AIE-induced anxiety-like behaviors. We infected the PVT neurons with an adeno-associated viral vector (AAV) expressing inhibitory DREADDs, hM4Di, under the control of a CaMKIIa promoter, leading to the selective expression of hM4Di in the projecting, excitatory PVT neurons [56–58] (Fig. 2a–c). In ex vivo electrophysiological recordings, we confirmed the application of the DREADDs ligand, JHU31760 (J60, 20 μM), effectively silenced the firing of PVT neurons expressing hM4Di (Fig. 2c). We then found that the administration of J60 (JHU37160, 0.3 mg/kg, i.p.) significantly increased the time spent in the center in the hM4Di-expressed AIE mice (Fig. 2e, two-way ANOVA, F(1,32) = 9.088, *P* = 0.0050, post-hoc AIE:Veh vs. AIE:J60: *p* = 0.0003), without the significant changes in total distance traveled during the OFT (Fig. 2f, post-hoc AIE:Veh vs. AIE:J60: *p* = 0.5427). In the EPM test, The chemogenetic inhibition of the PVT also increased the time spent in open-arms (Fig. 2h, two-way ANOVA, F(1,32) = 4.762, *P* = 0.0366, post-hoc AIE:Veh vs. AIE:J60: *p* = 0.0053). The chemogenetic inhibition of the PVT did not induce the increase in the time spent in the center (Fig. 2e, post-hoc CON:Veh vs. CON:J60: *p* = 0.9754) or open-arms (Fig. 2h, post-hoc CON:Veh vs. CON:J60: *p* = 0.9512). These data suggest that PVT inhibition could alleviate the anxiety-like behaviors in adult induced by AIE.

### Involvement of glutamatergic signaling between astrocytes and neurons in the AIE-induced adaptation of the PVT

**Fig. 3:**
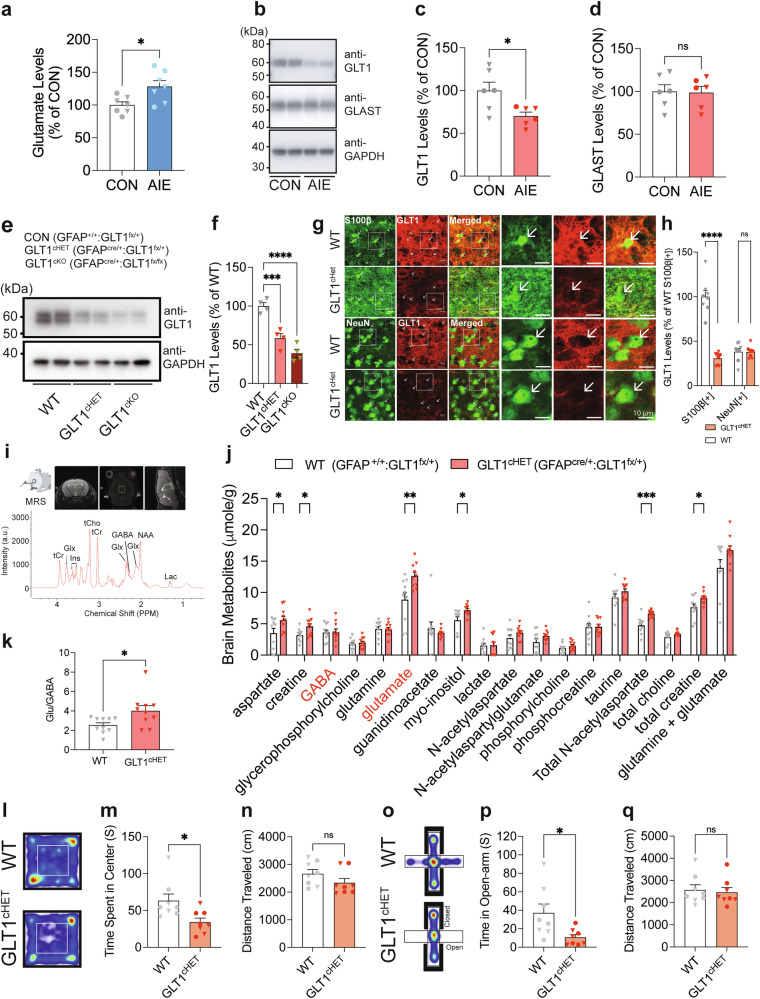
The expression of GLT1, an astrocytic glutamate transporter, is selectively reduced in AIE mice and GLT1 conditional knockdown induces anxiogenic phenotypes. **a** The quantification of glutamate levels in the dorsal thalamic area including PVT. Representative expression of GLT1 (as known as EAAT2), GLAST (as known as EAAT1), and GAPDH (**b**) and pooled data (**c**, **d**) of western blots showing that the GLT1 expression in the PVT of AIE mice is selectively reduced compared to that of the CON mice. Representative figures (**e**) and pooled data (**f**) confirming the reduction of GLT1 in the conditional GLT1 knockdown mice (GLT1^cHET^). Representative figures (**g**) and pooled data (**h**) showing the selective reduction of GLT1 in astrocytes, not in neurons of PVT. **i**–**k** Non-invasive magnetic resonance spectroscopy (MRS) measurement showing the increase in glutamate levels in the dorsal thalamic area, including PVT of GLT1^cHET^. **l**–**q** Representative traces (**l**, **o**) and pooled data (**m**, **n**, **p**, **q**) showing the anxiogenic profiles of GLT1^cHET^ in the open field test (**l**–**n**) and elevated plus maze test (**o**–**q**). Female (circles) and male (triangles). Data represented as mean ± SEM. *p < 0.05, **p < 0.01, ***p < 0.001, ****p < 0.0001. Fig. 3i, l, and o were created using BioRender.com.

Previous studies have demonstrated a hyper-glutamatergic state in brain, characterized by elevated extracellular glutamate, underlies increased susceptibility to chronic ethanol exposure and withdrawal-induced neurotoxicity and synaptic activity [59]. Specifically, astrocytic glutamate transporters, which have an critical role to scavenge the extra glutamate in the tripartite synapses, have been identified as a factor of brain adaptation that induces anxiety-like behaviors [60, 61] and those malfunctional adaptations are frequently observed after repeated ethanol exposure and withdrawal [62, 63]. Thus, to determine whether the capacity of glutamatergic signaling in the PVT is changed by AIE, we quantified glutamate levels and the protein levels of GLT1 and GLAST, the two major astrocyte-dominant transporters, in the PVT. First, we found the PVT tissue of AIE mice had an increase in glutamate levels compared to those of CON (Fig. 3a, Unpaired *t*-test, *t* = 2.757, df = 12, *p* = 0.0174, *N*_mice_ = 7/group). Interestingly, this increase in glutamate levels was coupled with a significantly reduced expression in GLT1 in the PVT tissue of AIE mice compared to that of CON (Fig. 3b, c, Unpaired *t*-test, *t* = 2.725, df = 10, *p* = 0.0214, *N*_mice_ = 6/group), while the GLAST expression remained unchanged (Fig. 3b, d, Unpaired *t*-test, *t* = 0.1174, df = 10, *p* = 0.9089, *N*_mice_ = 6/group). This data showed that AIE mice exhibited both hyper-glutamatergic conditions and reduced GLT1 expression in the PVT compared to control mice, suggesting that GLT1 may have a role in the AIE-induced glutamatergic pathophysiology in the PVT.

To assess whether the GLT1 reduction affects metabolites in the brain area, including glutamate in vivo, we measured the metabolites non-invasively using MRS in the mice with a GFAP-driven conditional heterozygous (cHET) reduction of GLT1 in the astrocytes (GLT1^cHET^, GFAP^cre/+^:GLT1^fx/+^; Fig. 3e–k and Supplementary Fig. 3). Conditional targeting was verified with GFAP-promoter driven GLT1 conditional knock-out mice (GLT1^cKO^, GFAP^Cre/+^:GLT1^fx/fx^; Fig. 3e, f, one-way ANOVA, F(2,9) = 34.20, *P* < 0.0001, *N*_mice_ = 4/group). Heterozygous reduction in GLT1 is sufficient to attenuate the GLT1 levels (post-hoc WT vs. GLT1^cHET^: *p* = 0.001) without significant changes in GLAST (Supplementary Fig. 3a–c, one-way ANOVA, F(2,9) = 0.3250, *P* = 0.7307, post-hoc WT vs. GLT1cHET: *p* = 0.9962) and xCT (Supplementary Fig. 3a, b, one-way ANOVA, F(2,9) = 3.492, *P* = 0.0754, post-hoc WT vs. GLT1^cHET^: *p* = 0.3683) levels in the PVT, similar to the changes by AIE (Fig. 3b–d). This heterozygous reduction in GLT1 also excludes the lethal phenotype shown in GLT1 cKO and global GLT1 KO [64, 65]. We confirmed that the reduction of GLT1 was in the astrocytes (Fig. 3g, h, two-way ANOVA, F(1,28) = 65.41, *P* < 0.0001, post-hoc S100β[+]:WT vs. S100β[+]:GLT1cHET: *p* < 0.0001, N_mice_ = 8/group), not in the neurons (post-hoc NeuN[+]:WT vs. NeuN[+]:GLT1cHET: *p* > 0.9999) of the PVT. The glutamate level in the thalamic area, including the PVT, was significantly reduced without changes in GABA levels, which induces the enhanced balance between glutamate and GABA ratio (Fig. 3i–k, Unpaired *t*-test, *t* = 2.497, df = 18, *p* = 0.0225, *N*_mice_ = 10/group). When we measured their behaviors in the OFT, the time spent in the center was significantly reduced in the GLT1^cHET^ mice compared to that in the CON mice (Fig. 3i–m, Unpaired *t*-test, *t* = 2.700, df = 14, *p* = 0.0173, *N*_mice_ = 8/group), with no change in distance traveled (Fig. 3n, Unpaired *t*-test, *t* = 1.472, df = 14, *p* = 0.1632). In the EPM test, the GLT1^cHET^ mice also showed the reduced time spent in open-arms (Fig. 3o–p, Unpaired *t*-test, *t* = 2.591, df = 14, *p* = 0.0213, *N*_mice_ = 8/group), with no change in distance traveled (Fig. 3q, Unpaired *t*-test, *t* = 0.3342, df = 14, *p* = 0.7432), suggesting the GLT1 reduction in astrocytes increase the thalamic glutamate levels and induces anxiety-like behaviors.

**Fig. 4:**
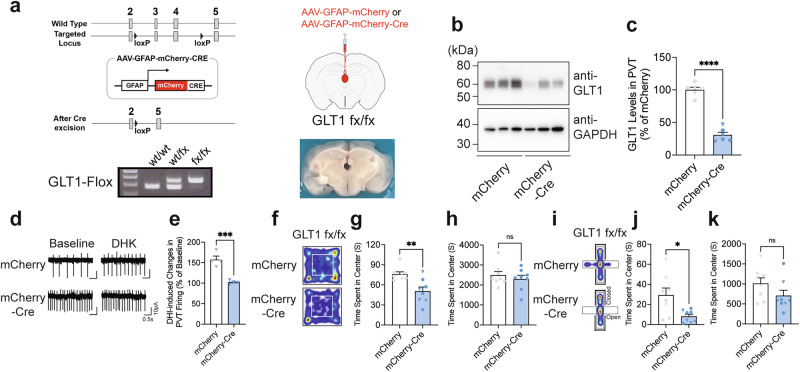
Region-specific conditional knock-out of GLT1 in the PVT mimics the anxiety-like behaviors induced by adolescent ethanol exposure. **a** Schematic drawing of Cre-loxP system for GLT1 cKO and genotyping of GLT1 wild-type and flox mouse. The representative figures (**b**) and pooled data (**c**) showing the reduction of GLT1 levels in the PVT after the GFAP-promoter driven expression of Cre in the PVT of GLT1 flox mouse. Representative traces (**d**) and pooled data (**e**) showing that the DHK-induced changes in neuronal firing were occluded in the PVT of GLT1 cKO. Representative traces (**f**, **i**) and pooled data (**g**, **h**, **j**, **k**) showing the effects of GLT1 cKO in the PVT astrocytes in the open field test (**g**, **h**) and elevated plus maze test (**i**–**k**). Female (circles) and male (triangles). Data represented as mean ± SEM. *p < 0.05, **p < 0.01, ****p < 0.0001. Fig. 4a, f, and i were created using BioRender.com.

Given the role of GLT1 reduction in anxiogenic phenotype, we sought to determine whether site-specific conditional knock-out of GLT1 in PVT induces the anxiogenic behavioral phenotype. Thus, we injected an AAV that has a capacity of a GFAP-promoter driven Cre recombinase expression in astrocytes into the PVT of ethanol naïve GLT1 flox mice (Fig. 4a, GLT1 cKO). The mice with the Cre recombinase expression showed a significant reduction in GLT1 in the PVT compared to mCherry controls (Fig. 4b, c, Unpaired *t*-test, *t* = 11.86, df = 10, *p* < 0.0001, *N*_mice_ = 6/groups). To further determine the functional adaptation of GLT1 cKO in the PVT, we compared the effects of dihydrokinic acid (DHK), a selective GLT1 inhibitor in the PVT neurons of WT and GLT1cKO mice (Fig. 4d, e). Bath application of DHK (200 μM) significantly increased the neuronal spiking of the PVT neurons in the WT, while the changes by the bath-applied DHK in the PVT neurons were significantly reduced in the GLT1 cKO (Unpaired *t*-test, *t* = 6.166, df = 6, *p* = 0.0008, N_cell_ = 4/group [4mice/group]). In the OFT, the mice of GLT1 cKO in the PVT showed the decrease in the time spent in the center (Fig. 4g, Unpaired *t*-test, *t* = 3.471, df = 14, *p* = 0.0037, *N*_mice_ = 8/group) without significant changes in distance traveled (Fig. 4h, Unpaired *t*-test, *t* = 0.7261, df = 14, *p* = 0.4797). In the EPM test, the mice also showed the reduced time spent in open-arms (Fig. 4j, Unpaired *t*-test, *t* = 2.837, df = 14, *N*_mice_ = 8/group) without changes in distance traveled (Fig. 4k, *t* = 1.539, df = 14, *p* = 0.1461, *N*_mice_ = 8/group), suggesting that selective knock-out of GLT1 in the PVT astrocytes induces the anxiogenic phenotype.

### Selective rescue of GLT1 expression in the PVT of AIE mice ameliorate the AIE-induced anxiety-like behaviors

**Fig. 5:**
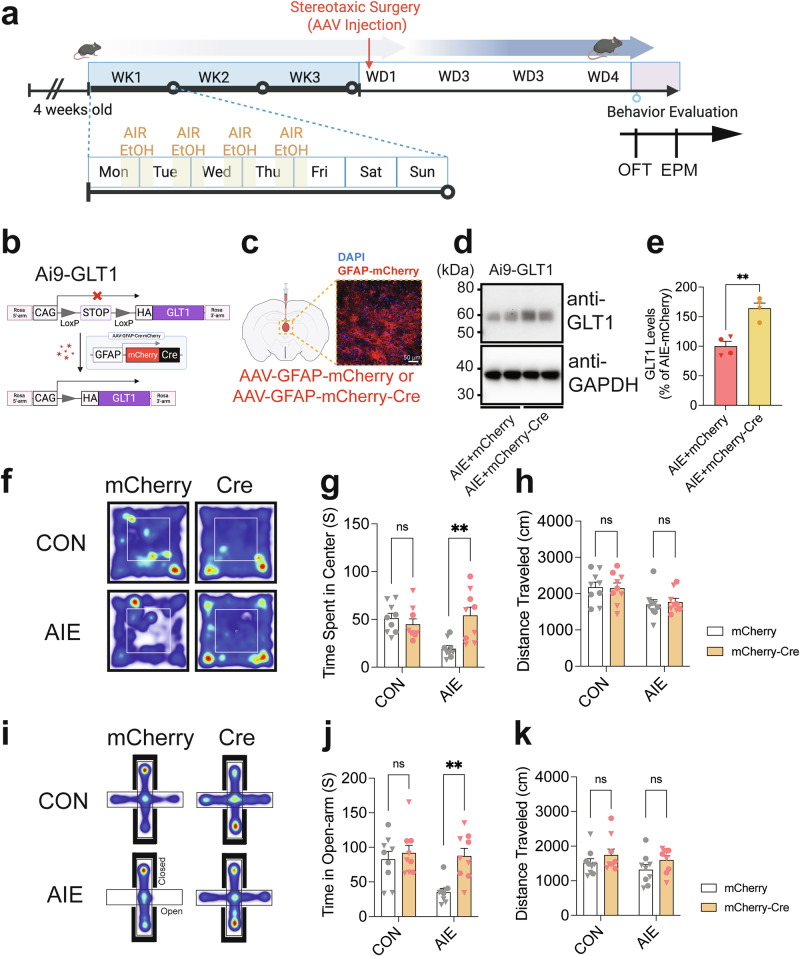
Rescue of GLT1 expression in the astrocytes of the PVT ameliorates the AIE-induced anxiety-like behavior. Diagram of experimental schedules (**a**) and transgenic approach to overexpress GLT1 in the astrocytes of PVT in a cell-type specific manner (**b**). Diagram and representative expression (**c**), representative blots (**d**), and pooled data (**e**) showing the increased GLT1 expression in the PVT after the AAV-induced Cre expression in the PVT of the Ai9-GLT1 AIE mice. **f**–**k** Representative traces (**f**, **i**) and pooled data (**g**, **h**, **j**, **k**) showing that the astrocytic GLT1 rescue in the PVT ameliorates the AIE-induced anxiety-like behavior in the open field test (**f**–**h**) and elevated plus maze test (**i**–**k**). Female (circles) and male (triangles). Data represented as mean ± SEM. **p < 0.01. Fig. 5a, b, c, f, and i were created using BioRender.com.

Because AIE reduced the GLT1 expression in the PVT and induced the hyper-glutamate levels, which was accompanied with anxiogenic phenotypes, we sought to determine whether restoring GLT1 expression in the PVT would rescue the behavioral adaptation induced by AIE. We injected an AAV to express Cre recombinase in astrocytes using the GFAP promoter into the PVT of the AIE experienced transgenic Ai9-GLT1 mice (Fig. 5a). The Ai9-GLT1 mice have a capacity to delete the stop-codon in a Cre-recombinase activity dependent manner, leading a continuous increase in GLT1 expression (Fig. 5b–e, Unpaired *t*-test, *t* = 5.398, df = 6, *p* = 0.0017, *N*_mice_ = 4/group). In the OFT, the mice restoring astrocyte GLT1 in the PVT rescued the reduced time spent in the center by AIE experience (Fig. 5f, g, two-way ANOVA, F(1,32) = 11.71, *P* = 0.0017, post-hoc AIE:mCherry vs. AIE:mCherry-Cre: *p* = 0.0015), without changes in distance traveled (Fig. 5h, two-way ANOVA, F(1,32) = 0.1235, *P* = 0.7275, post-hoc AIE:mCherry vs. AIE:mCherry-Cre: *p* = 0.9827). In the EPM test, the PVT GLT1 rescued mice also showed recovered time spent in the open arms, which was reduced by AIE (Fig. 5i, j, two-way ANOVA, F(1,32) = 4.723, *P* = 0.0373), without changes in distance traveled (Fig. 5k, two-way ANOVA, F(1,32) = 0.03659, *P* = 0.8495, post-hoc AIE:mCherry vs. AIE:mCherry-Cre: *p* = 0.4941). These data suggest that rescue of GLT1 expression in the PVT of AIE mice ameliorated the anxiogenic phenotype as observed in AIE mice.

Taken together, our findings support the hypothesis that adolescent repeated ethanol exposure alters the capacity for glutamatergic communication between astrocytes and neurons in the PVT, leading to the anxiogenic phenotypes in adult, at least partly, via the astrocyte glutamate transporter, GLT1.

## Discussion

In the present study, we provide comprehensive evidence of apparent anxiety-like behaviors in adult mice that were exposed ethanol repeatedly in their adolescent period. While the PVT neuronal activity and glutamatergic signals are enhanced, the GLT1 expression is selectively reduced in the PVT. Importantly, chemogenetic inhibition of the PVT neurons or transgenic restoration of GLT1 in the PVT ameliorate the anxiety-like behaviors induced by adolescent repeated ethanol exposure. These findings suggest that GLT1 in the PVT plays a significant role in the brain adaptation for the anxiety susceptibility in adulthood after adolescent intermittent repeated ethanol exposure.

### PVT hyperactivity and astrocytic GLT1 dysregulation in anxiety-like behaviors by adolescent intermittent ethanol exposure

Similar to the previous reports of the anxiogenic effects of ethanol on rodents [16, 17, 66–69], we described anxiety-like behaviors in mice withdrawn from repeated ethanol exposure during the adolescent period, as shown by the reduced time spent in the center of an open field, and in the open arms of an elevated plus maze, as well as the less time in the light illuminated area. The total distance traveled remained unchanged, indicating the adolescent repeated ethanol exposure did not significantly impact the locomotor activity in our model. Notably, we observed the adaptation of cellular activities in the PVT. Although the PVT has been receiving increasing attention to be linked with anxiety-related behaviors, especially behavioral malfunction induced by adverse experiences during early in life [20], its role in the context of withdrawal from adolescent repeated ethanol exposure was unknown. We found that PVT neurons in ethanol-withdrawn adults (AIE) had a significantly higher spontaneous firing rates, suggesting a possible contribution of PVT hyperactivity to the increased anxiety levels. This possibility was supported by our data showing that selective inhibition of PVT neurons by chemogenetic approach or transgenic upregulation of GLT1 in the PVT mitigated elevated anxiety-like phenotypes associated with the ethanol withdrawal. Searching for the underlying cellular and molecular mechanisms, we found that the PVT neurons of AIE mice had an enhanced level of glutamate and glutamate/GABA ratio, compared to CON mice and this is accompanied with the reduction of GLT1 levels in the PVT, suggesting that GLT1 dysregulation may contribute to the observed increased activities of PVT neurons in the AIE mice. These data support a functional relationship between anxiety-like behaviors and the activities of PVT. The PVT is one of the main upstream inputs to the amygdala, a region recognized as a central hub within anxiety-processing networks and previously reported to undergo adaptive changes after long-term withdrawal reaching to adulthood from AIE exposure [69]. Recent anatomical and functional studies, dissecting the roles of PVT in an anterior-posterior axis and amygdala in a medio-lateral axis, demonstrate that the posterior PVT (pPVT) maintains robust projections to the central amygdala (CeA) and plays a critical role in anxiety modulation [70]. Compared to the anterior part of PVT (aPVT), the pPVT exhibits greater involvement in pain-induced anxiety processing, which aligns with our findings regarding PVT hyperactivity in adapted anxiogenic states. Given emerging evidence for functional diversity along the anterior-posterior axis of the PVT, where distinct subregions differently modulate amygdala sub-regional populations and anxiety-related behaviors, future investigations should examine how PVT subregional astrocyte populations influence downstream amygdala circuitry and broader multiple regional anxiety-networks. Such studies would advance our understanding of how local events by astrocyte-neuron interactions contribute to whole-brain homeostatic regulation.

### Possible molecular mechanisms in GLT1 regulation

The observation of prolonged brain adaptation, including the reduction of GLT1 protein expression in AIE mice may be caused by changes in the factors that affect the corresponding gene expression. For example, the transcription factor, Pax6, has been identified as a common mechanism of regulating GLT1 expression, without expression on the levels of another astroglial glutamate transporter, GLAST, by binding within the upstream of the GLT1 translation start site [71], which is also aligned with our findings in the PVT. Indeed, the Pax6 is expressed early in development, predominantly in a few body parts, including the brain [72], and has an essential role for early animal development [73, 74]. While the expression of Pax6 is reduced after ethanol exposure [75], Pax6 overexpression rescued alcohol-induced cellular dysfunction [76]. In addition, multiple epigenetic mechanisms via microRNAs (miRNAs), which are short non-coding RNAs modulating mRNA translation, have been suggested to contribute to the brain adaptation due to alcohol exposure during adolescence, which increase the susceptibility in adulthood to alcohol use and anxiety [42, 66, 69, 77, 78]. Thus, given the role of miRNAs and epigenetic impacts in the regulation of transcription factors, including Pax6 [79–81], it is intriguing to investigate the mechanisms how the synergistic reciprocal epigenetic modulations with related transcription factors affect the selective changes in GLT1 of the PVT by the adolescent repeated ethanol.

### Further directions: cell types, circuits, and sex differences

Although we did not identify the cell type we recorded in the current study, it has been known that the neurons in the PVT are majorly glutamatergic [82–84]. Recently, accumulative observations have provided the additional information there are diverse neuronal subpopulations in the PVT according to significant transcriptomic variance corresponding to the anterior-posterior axis [85, 86], which may contribute to difference multi-brain regional connectivity and diverse behaviors. Importantly, not only Central and Basolateral Amygdala (CeA, BLA) by direct and indirect connection, PVT also has been well known to have a role of pivotal region for anxiety-network including the bed nucleus of the stria terminalis (BNST) and Nucleus Accumbens (NAc) [25], where are the largest cluster of anxiety-network in the rodent and primate brains, participating in mediation of generalized anxiety and related arousal behaviors [87, 88], and environment-raised anxiety and reward motivation behaviors [89, 90], respectively. However, the functional significance of the various sub-connections from the PVT during ethanol withdrawal, especially adapted by the repeated exposure to ethanol during adolescent period, remains poorly addressed. Thus, future studies are also needed to clarify which cell-types and circuits are more significantly impacted by adolescent repeated ethanol exposure in an age-dependent manner. In addition, given the role of PVT in arousal and vigilance-linked behaviors and the connection between anxiety and hyperarousal, it will be intriguing to investigate the role of PVT GLT1 in arousal-related behavioral patterns, including social behaviors [25, 27, 91] and sleep-wake cycles [92, 93]. One limitation of our study is the exclusion of sex differences by the use of both male and female mice. Although previous studies showed that adult anxiety induced by AIE is observed in a sex independent manner [41, 42], many of the psychiatric and neurological disorders induced by AIE are manifest only observed in one sex [13]. Thus, age- and sex- dependent evaluation need to be further studied as well.

Our data here suggests that GLT1 downregulation-induced PVT hyperactivity in adults exposed to adolescent repeated ethanol exposure contributes to anxiety-like behaviors. This study implies the importance of glutamatergic homeostatic balance through GLT1 in the adult behavioral susceptibility induced by the early life adverse experience.

## Supplementary information

Supplementary Figures

Supplementary Table 1

## Data Availability

All data are available from the authors upon reasonable request.
