# Supplementary material for: Glutamatergic dysfunction of astrocytes in paraventricular nucleus of thalamus contributes to adult anxiety susceptibility in adolescent ethanol exposed mice
Source: Neuropsychopharmacology. 2025 Oct 13;51(4):778–90. doi: 10.1038/s41386-025-02264-3 (PMC12646275; doi:10.1038/s41386-025-02264-3)
Supplement: Supplementary file 1 — Supplementary Figures [file 41386_2025_2264_MOESM1_ESM.docx]

Supplementary Information


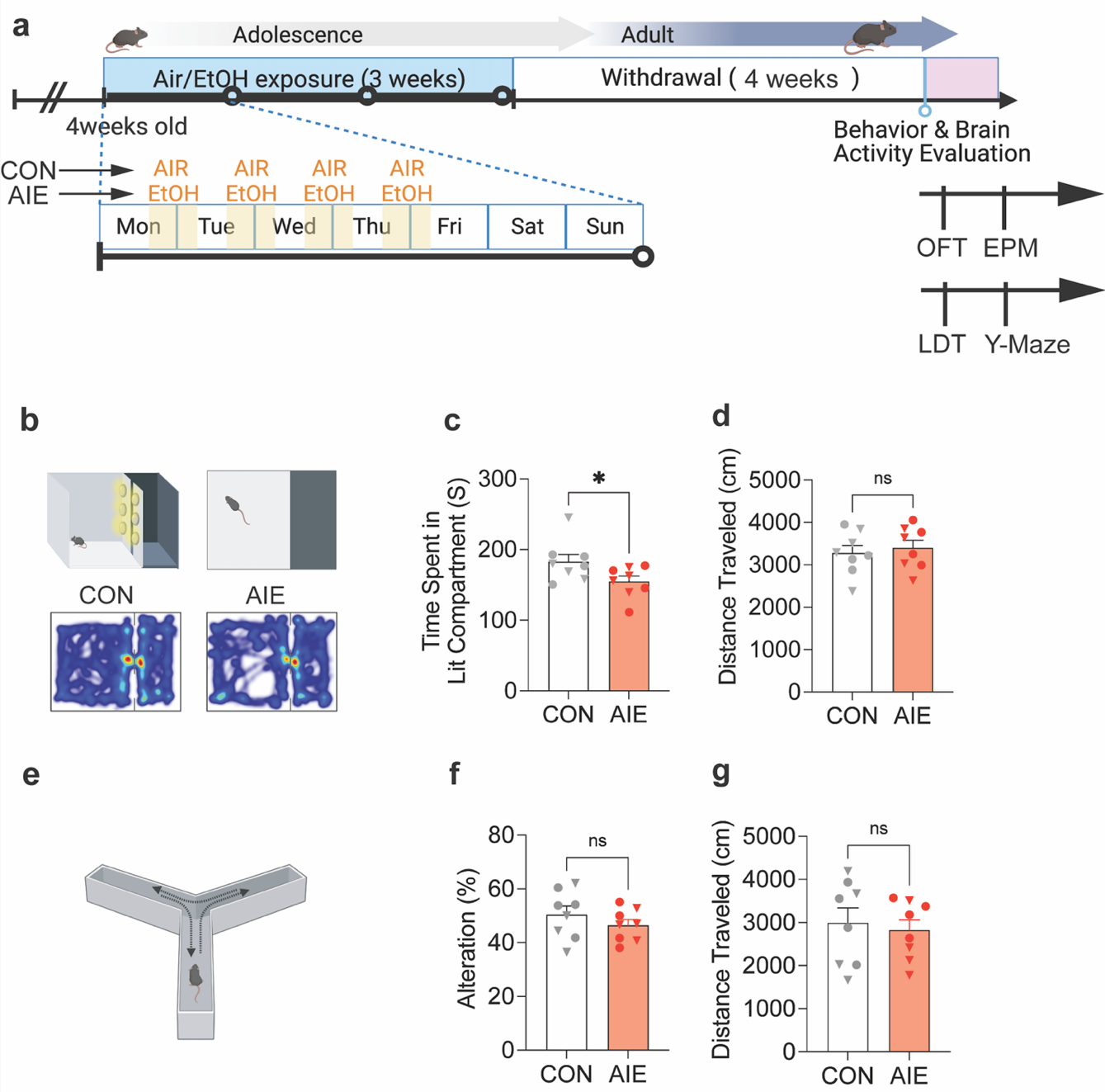


**Supplementary Fig. 1.** Adolescent repeated ethanol exposure (AIE) induces anxiety-like behaviors, but not working memory deficits, in adult mice. (a) Diagram of experimental schedules. (b-d) pooled data showing the times spent in lit compartment (c) and distance traveled (d) in the light-dark box test. (e-g) pooled data showing the percentage of complete alteration (f) and distance traveled (g) in y-maze test. Data represented as mean ± SEM. *p<0.01. Sumpplementary Figure 1a, 1b, and 1e were created using BioRender.com.


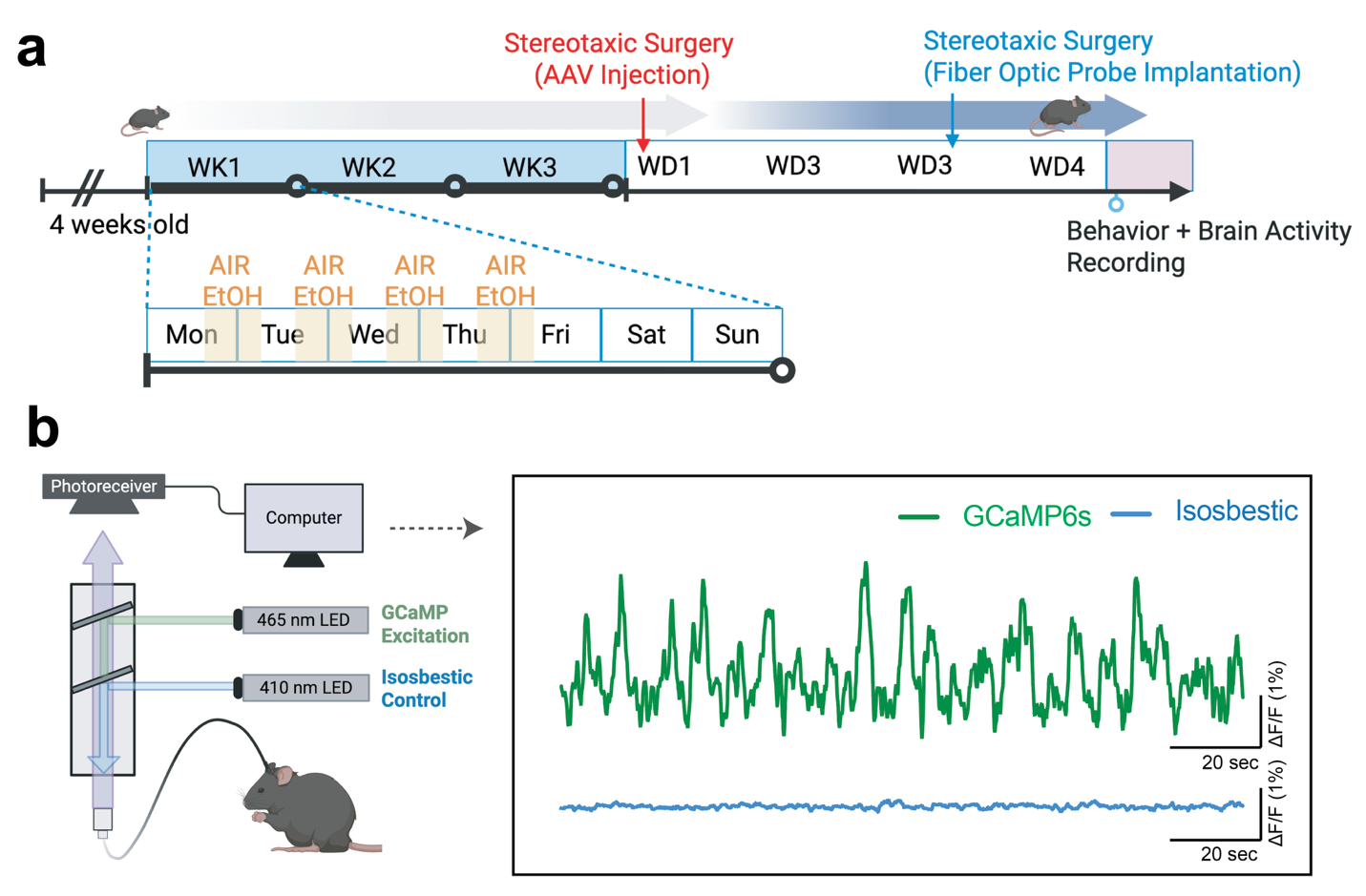


**Supplementary Fig. 2.** Fiber-photometry experiment. (a-b) Diagram of experimental schedules (a) and fiber-photometry system with the example traces of GCamP6s-based signals and isosbestic control (b). Supplementary Figure 2a and 2b were created using BioRender.com.


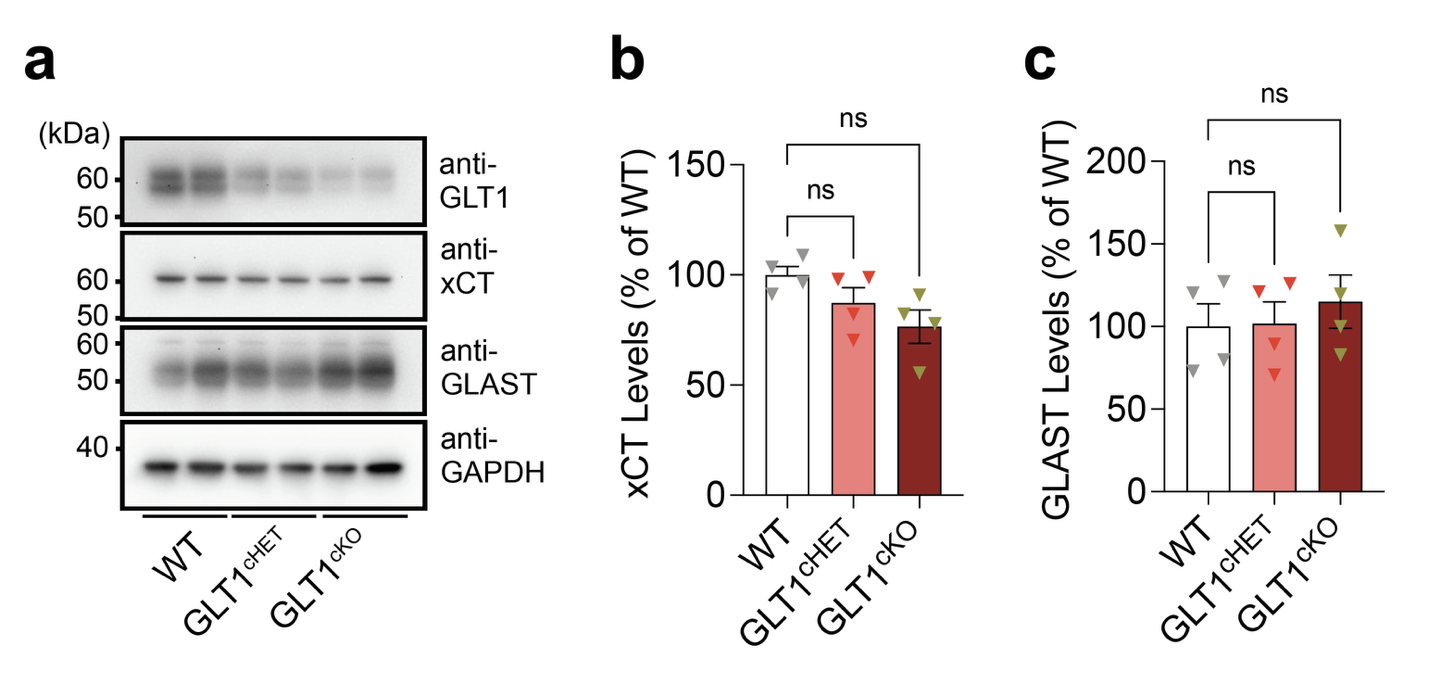


**Supplementary Fig. 3.** GLT1 conditional knockdown does not induce significant changes in xCT or GLAST levels. Representative blots (a) and pooled data (b-c) showing the levels of xCT and GLAST in the PVT. Data represented as mean ± SEM.
